# Maturation of Cognitive Control: Delineating Response Inhibition and Interference Suppression

**DOI:** 10.1371/journal.pone.0069826

**Published:** 2013-07-23

**Authors:** Christopher R. Brydges, Mike Anderson, Corinne L. Reid, Allison M. Fox

**Affiliations:** 1 Neurocognitive Development Unit, School of Psychology, University of Western Australia, Perth, Western Australia, Australia; 2 School of Psychology, Murdoch University, Perth, Western Australia, Australia; University of Rome, Italy

## Abstract

Cognitive control is integral to the ability to attend to a relevant task whilst suppressing distracting information or inhibiting prepotent responses. The current study examined the development of these two subprocesses by examining electrophysiological indices elicited during each process. Thirteen 18 year-old adults and thirteen children aged 8–11 years (mean = 9.77 years) completed a hybrid Go/Nogo flanker task while continuous EEG data were recorded. The N2 topography for both response inhibition and interference suppression changed with increasing age. The neural activation associated with response inhibition became increasingly frontally distributed with age, and showed decreases of both amplitude and peak latency from childhood to adulthood, possibly due to reduced cognitive demands and myelination respectively occurring during this period. Interestingly, a significant N2 effect was apparent in adults, but not observed in children during trials requiring interference suppression. This could be due to more diffuse activation in children, which would require smaller levels of activation over a larger region of the brain than is reported in adults. Overall, these results provide evidence of distinct maturational processes occurring throughout late childhood and adolescence, highlighting the separability of response inhibition and interference suppression.

## Introduction

Cognitive control refers to the group of processes required to resist interference from distracting stimuli or prepotent automatic responses, whilst attending to task-relevant information [1], [2]. These inhibitory processes are often considered to be important components of intelligence [3]–[5], as well as affecting an individual’s ability to function in everyday life [6]. In the past 10–15 years, interest in how inhibition is associated with other executive functions (especially shifting and updating of working memory) has been a particular area of focus [7]–[9]. However, although several theorists have proposed that subprocesses of inhibition should be considered as related yet separable, only a minimal amount of research has examined the validity of these claims (but see [10]–[12]).

The present study focuses on response inhibition (the suppression of a prepotent or automatic behavioural response) and interference suppression (the ability to control for distracting stimuli or information due to stimulus competition; 13). Nigg proposed a taxonomy of inhibition, of which response inhibition and interference suppression are two distinct yet related processes [13]. Other prominent theories of inhibition [14]–[16] may use different terminology for these constructs; however, each of these theories converges upon the notion that inhibition refers to several separate but interrelated processes, rather than a singular construct.

A recent study by Brydges, Clunies-Ross et al. reported electrophysiological evidence in support of the separability of response inhibition and interference suppression in young adults [10]. Participants completed a hybrid Go/Nogo flanker task whilst having an electroencephalogram (EEG) recorded. The N2 event-related potential (ERP), which is commonly associated with inhibition on both Go/Nogo and flanker tasks [17]–[21], was analysed between the incongruous condition (measuring interference suppression) and the Nogo condition (measuring response inhibition). Two major findings were reported: first, the N2 peak associated with each process was maximal at different scalp sites, and the peak latency differed significantly between conditions. Specifically, the N2 elicited in the incongruous condition was maximal at the central midline site, and had a significantly longer latency than the N2 elicited in the Nogo condition, which was maximal at the frontal midline site. From this, it was suggested that these topographical differences were due to these two processes originating from different neural regions or that a common set of generators differentially contribute to each process. Additionally, the latency difference suggests that interference suppression may require additional cognitive processing over and above that required for successful response inhibition [16], [22], providing further evidence for the separability of the proposed subprocesses of inhibition.

The maturation of inhibitory processes and other executive functions is of critical importance in children, particularly in educational settings [23]. Previous research has found marked improvements on behavioural measures of inhibition throughout childhood and, in some cases, into mid-adolescence [24]–[26]. Huizinga et al. reported improved performance on both a stop-signal task and a flanker task between groups of children aged 7, 11, and 15 years respectively, suggesting that there may be some common developmental process that leads to the improvement of both response inhibition and interference suppression.

From a neuroimaging perspective, Bunge et al. [11] examined the maturation of these two processes by using functional magnetic resonance imaging (fMRI) to record neural activity whilst adults and children aged 8–12 years completed a hybrid Go/Nogo flanker task. It was reported that children displayed activation of posterior regions of the brain during successful response inhibition, whereas prefrontal regions were activated in adults. During successful interference suppression, prefrontal regions were activated for both groups; however, only the left hemisphere was activated in children, whilst only the right hemisphere was activated in adults. Hence, it is apparent that neural development of cognitive control occurs at a significant rate through late childhood and adolescence [27], [28]. One possible drawback of the task used by Bunge et al., however, is that the flanker stimuli acted as cues to inhibit responses in the Nogo condition of their task. That is, in the conditions that required a response, the flanker stimuli were meant to be ignored, but participants were required to actively attend to them in the Nogo condition. This could have changed the manner in which participants processed the incongruous stimuli, supported by the low error rates in this condition.

No previous research has used ERPs to simultaneously examine the maturation of response inhibition and interference suppression. When examining response inhibition, Johnstone et al. [29] recorded EEG data whilst groups of children, and young and older adults completed a Go/Nogo task, and found that N2 peak latency significantly decreased from childhood to adulthood, perhaps due to myelinisation occurring during this period of childhood, hence increasing neural speed [30]. N2 peak amplitude also significantly decreased with age, due to greater activation of regions of the prefrontal cortex in children than in adults [31]. Additionally, Jonkman et al. [32] reported that the medial frontal cortex (near the anterior cingulate cortex) is activated during response inhibition and associated with the N2 in both children and adults. There is a scarcity of literature examining the electrophysiological development of interference suppression through childhood; however, Rueda et al. [33] found a significant decrease of N2 peak latency between four year-old children and adults during completion of a child-friendly flanker task. However, the amplitude of the N2 was very small in the group of children, and became larger in the adult group. It was claimed that these differences are neural evidence of the incomplete development of interference suppression processes in children.

The aim of this study was to examine the maturation of response inhibition and interference suppression simultaneously from an electrophysiological perspective. It was hypothesised that the results observed by Brydges et al. [10] would be replicated in the adult sample. Specifically, the N2 associated with response inhibition have a shorter latency and be more frontally distributed than that of the N2 associated with interference suppression. Additionally, it was hypothesised that the site of maximal amplitude of the N2 ERP associated with response inhibition would become increasingly frontal between childhood and adulthood [11], [28], and that the N2 amplitude and peak latency would both significantly decrease with age [29]. Furthermore, it was hypothesised that there would be no change in the site of maximal amplitude of the N2 ERP associated with interference suppression between children and adults. However, based on the results of Rueda et al. [33] there would be a significant increase in the amplitude of the N2, and a significant decrease of peak latency, with age. In addition to ERP analyses, source localisation was conducted on each group and condition, and was expected to display further evidence of different neural generators between conditions.

## Methods

### Ethics Statement

Approval for the study was provided by the Human Research Ethics Office of The University of Western Australia (both groups) and by the Princess Margaret Hospital Ethics Committee (child group). All adult participants and parents/guardians of the child participants provided written informed consent.

### Participants

Twenty six participants were recruited and split into two groups of thirteen. The group of typically developing children were aged 8–11 years (*M* = 9.77 years; 9 females and 4 males), and the adults (8 females and 5 males) were all aged 18 years. Children were recruited through Project K.I.D.S. (Kids’ Intellectual Development Study), a research program examining the cognitive, social, and emotional development of children run by the Neurocognitive Development Unit of the School of Psychology of the University of Western Australia. The young adults were first-year undergraduate psychology students who participated in order to partially fulfil course requirements. Both groups completed the task as part of a larger test battery.

### Materials

**Figure 1:**
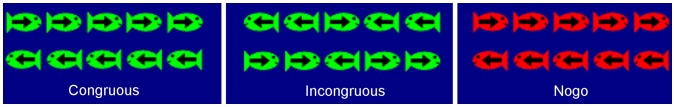
The six stimuli used in the present experiment (taken from Brydges, Clunies-Ross, et al., 2012).

The same hybrid Go/Nogo flanker task used by Brydges et al. [10] was used in this study. Each stimulus consisted of five fish presented on a blue background. An arrow on the body of the fish specified direction and the target was the central fish. Participants were instructed to press a response button on a keyboard (red felt patches on the ‘Z’ and ‘/’ keys) analogous to the direction of the central fish. The task had three conditions: in the congruent condition (.5 probability), the fish were green and all facing the same direction. In the incongruent condition (.25 probability), the fish were also green, however the flankers faced the opposite direction to the central target. In the Nogo condition (.25 probability), the fish were congruent but were all red, the participant was required to not respond. Each fish subtended.9° horizontally and.6° vertically, with.2° separating each fish (see Figure 1). Stimuli were presented in random order for 300 ms with a 2,000 ms inter-stimulus interval. The task was presented to the children as a game in which the participants had to feed the hungry central fish. Speed and accuracy were equally emphasized. Eight practice trials were administered to ensure the participants understood the task requirements. A total of 176 trials were subsequently presented in one block.

### Electrophysiological Acquisition

The EEG was continuously recorded using an Easy-Cap™. Electrodes were placed at 33 sites based on Easy-Cap montage 24 (see http://www.easycap.de/easycap/e/products/products.htm for more details). Eye movements were measured with bipolar leads placed above and below the left eye. The EEG was amplified with a NuAmps 40-channel amplifier, and digitized at a sampling rate of 250 Hz. Impedances were below 5 kΩ prior to recording. During recording, the ground lead was located at AFz and the right mastoid was set as reference, and a common averaged reference was calculated offline. Scan 4.3 was used to conduct the ERP processing. Offline, the EEG recording was digitally filtered with a 1–30 Hz zero phase shift band-pass filter (12 dB down). The vertical ocular electrodes enabled offline blink reduction according to the standard algorithm proposed by Semlitsch et al. [34].

### Data Analysis

Epochs encompassing an interval from 100 ms prior to the onset of the stimulus and extending to 1000 ms post-stimulus were extracted and baseline corrected around the pre-stimulus interval. Epochs containing artifacts larger than 150 µV or where an incorrect behavioural response was committed were excluded from the ERP average. Difference waveforms were then calculated by subtracting the individual ERP average elicited following presentation of the congruent stimuli from the ERP average elicited following presentation of the incongruent stimuli and the Nogo stimuli. We calculated the interval over which the N2 inhibition effect was significant by comparing the amplitude of the difference waveforms at each time point from 100–550 ms against a mean value of zero. To control for the number of comparisons conducted, we required a successive sequence of 11 statistically significant values based on an autocorrelation of 0.9 and graphical threshold of 0.05, as detailed by Guthrie and Buchwald [35]. In the group of children, the incongruous N2 effect was not significant at Fz, FCz, or Cz. In the Nogo condition, the N2 effect was significant at Cz between 388–464 ms only. In the adult group, the incongruous waveform was significant at Fz, FCz, and Cz, during respective latencies of 312–360, 304–380, and 296–388 ms. These latency windows were averaged to 304–376 ms for analyses. In the Nogo condition, the N2 waveform was significant at Fz (128–180 ms and 224–264 ms) and FCz (136–180 ms). However, upon examination of the difference waveforms, it was apparent that the two early waveforms at these sites were N1 peaks, and were excluded from analyses.

Source localisation analyses were conducted on each condition in the adult group using BESA 5.1. The same analyses were attempted on the group of children; however, the observed results were inadmissible. Instantaneous dipole models were computed on grand average ERP difference waveforms of each condition within the latency windows mentioned previously. A four-shell ellipsoidal head model with default values of bone thickness (7.0 mm) and conductivity (0.0042) was used for analyses. Dipole pairs were fitted with locations and orientations constrained to be mirror-symmetrical. Source models were computed in a 12 ms window around the N2 difference peak latency at the site of maximal amplitude for each of the conditions (276 ms at Fz for the Nogo – congruous difference waveform, and 352 ms at FCz for the incongruous – congruous difference waveform). Source models were considered acceptable if they explained at least 95% of the variance, and were stable across different starting points. The reported solutions were stable across different starting positions.

A mixed design ANOVA with scalp site (Fz, FCz, Cz) as a repeated measures factor was conducted on the mean amplitudes extracted. Latency and amplitude of the N2 effect were quantified for peaks within a 212–464 ms latency interval at the site of maximal amplitude only. This window was chosen to capture the intervals identified in difference waveform analyses for both conditions in each age group, and to ensure the maximum point was identified in each participant’s waveform.

## Results

### Behavioral Results

Descriptive statistics of behavioural results are presented in Table 1. A 2×2 mixed design ANOVA with reaction time (congruous and incongruous) as a repeated measures factor found that performance was impaired in the incongruous condition in comparison to the congruous condition (*F*(1, 24) = 57.22, *p*<.001, η_p_^2^ = .70). Additionally, performance significantly improved with age (*F*(1, 24) = 28.23, *p*<.001, η_p_^2^ = .54). However, the interaction between age group and condition was not significant (*F*(1, 24) = 0.38, *p* = .54, η_p_^2^ = .02).

**Table 1:** Descriptive statistics of behavioural measures between groups (means, with standard deviations in parentheses).

Age group	Congruous		Incongruous		Nogo
	Reaction Time	% correct	ReactionTime	%correct	% correct
Children	637 (184)	.91 (.06)	705 (167)	.79 (.05)	.88 (.10)
Adults	379 (35)	.97 (.04)	437 (51)	.85 (.10)	.98 (.03)

### ERP Results

**Figure 2:**
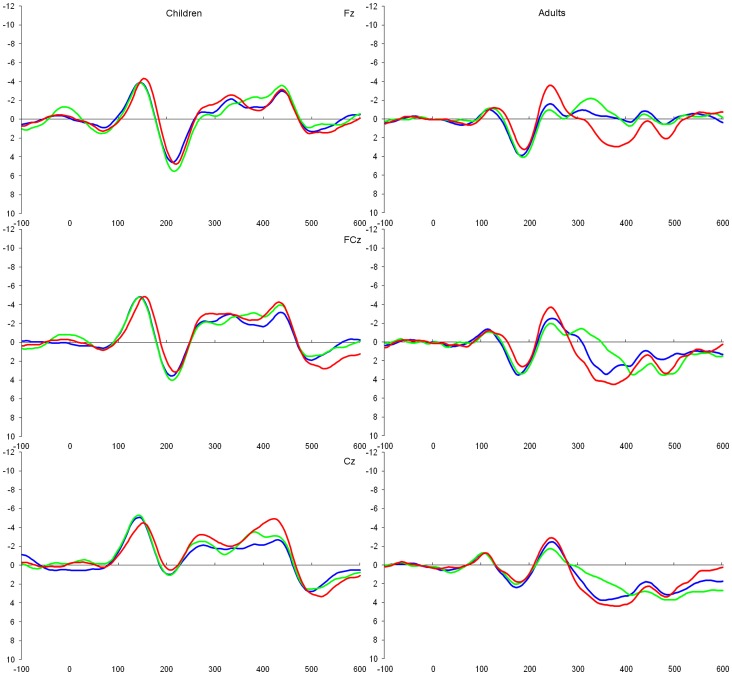
Stimulus-locked grand average ERP waveforms in response to congruous (blue), incongruous (green), and Nogo (red) stimuli with the amplitude (µV) as the y-axis and time (ms) as the x-axis. Time 0 represents stimulus onset.

**Figure 3:**
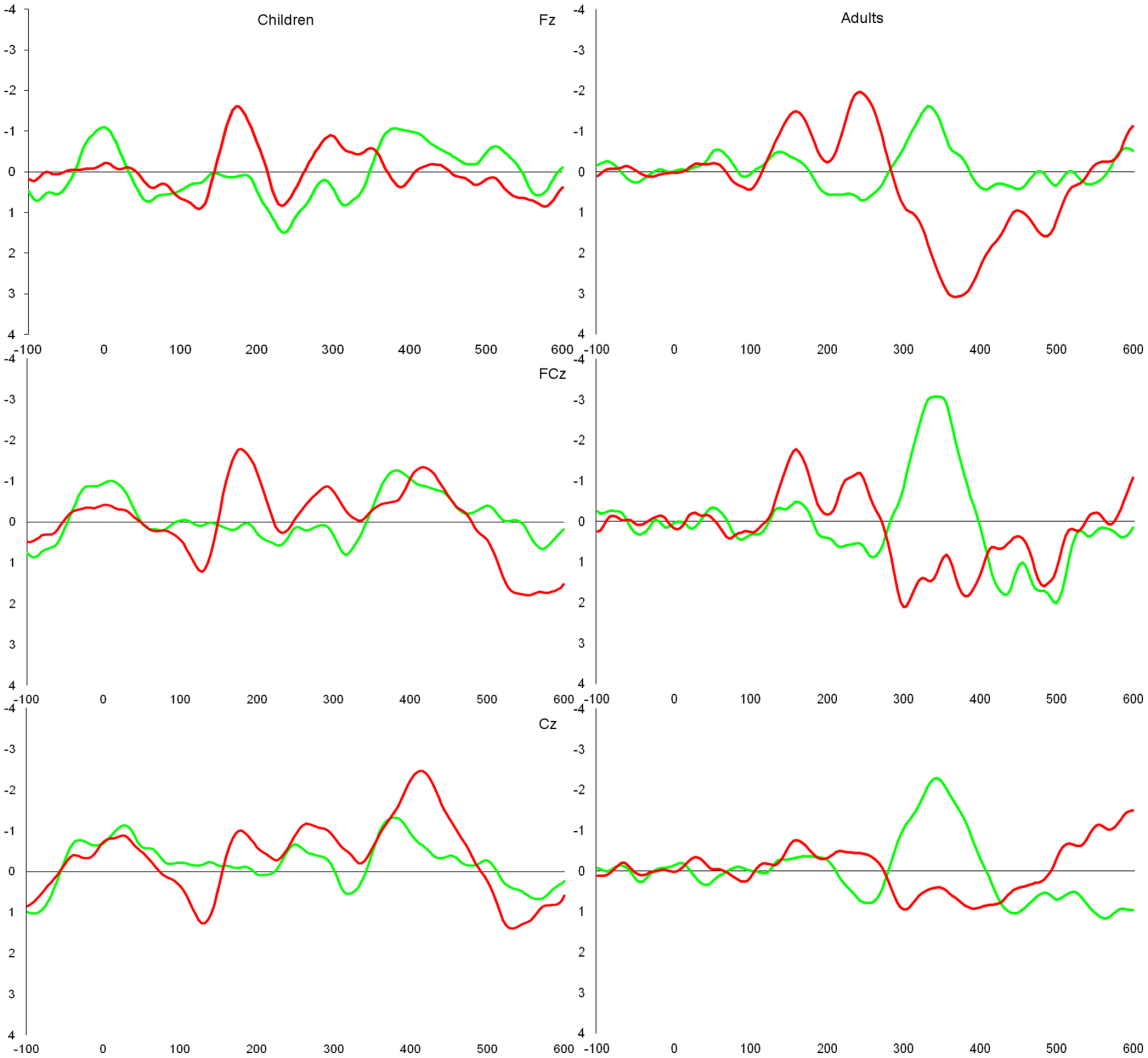
Grand-averaged difference waveforms computed as the incongruous – congruous waveform (green) and Nogo – congruous (green) with the amplitude (µV) as the y-axis and time (ms) as the x-axis. Time 0 represents stimulus onset.

The mean N2 amplitude of the incongruous – congruous difference waveform of one adult participant was considered an extreme value (greater than 3 *SD*s from the mean), and was replaced with a value 3 *SD*s from the mean for statistical analyses. Figure 2 shows the stimulus-locked grand averaged waveforms for each condition between age groups, and Figure 3 shows the difference waveforms computed by subtracting the ERPs elicited to the congruous stimuli from each of the other two waveforms. The amplitudes and latencies of the N2 peak identified in the difference waveforms are summarised in Table 2.

**Table 2:** N2 amplitude and latency summary statistics between groups (means, with standard deviations in parentheses).

Group	Condition	Site	N2 MA	N2 PkA	N2 PkL
Children	IS – CS	Fz	–	–	–
		FCz	–	–	–
		Cz	–	–	–
	NG – CS	Fz	0.00 (1.16)		
		FCz	−0.93 (1.78)		
		Cz	−1.86 (1.93)	−4.08 (1.79)	352.00 (64.06)
Adults	IS – CS	Fz	−1.04 (1.19)		
		FCz	−2.37 (2.11)	−3.35 (2.00)	350.46 (36.90)
		Cz	−1.80 (0.94)		
	NG – CS	Fz	−1.70 (1.80)	−2.66 (1.86)	275.69 (80.22)
		FCz	−0.90 (2.07)		
		Cz	−0.41 (1.43)		

The results of Brydges et al. [10] were generally replicated: the negativity observed in the Nogo – congruous difference waveform was more frontally distributed (Fz>FCz>Cz) than that observed in the incongruous – congruous difference waveform (FCz>Cz>Fz), as evidenced by a significant interaction between scalp site and condition (*F*(2, 24) = 3.96, *p* = .033, η_p_^2^ = .25). Additionally, the peak latency of the incongruous – congruous difference waveform was significantly longer than that of the Nogo – congruous difference waveform (*F*(1, 12) = 8.24, *p* = .014, η_p_^2^ = .41).

The negativity observed in the Nogo – congruous difference waveform did not produce a significant main effect of electrode site (*F*(2, 48) = 0.47, *p* = .63, η_p_^2^ = .02) or of age group (*F*(1, 24) = 0.18, *p* = .90). However, a significant interaction between site and age group was observed (quadratic trend; *F*(1, 24) = 19.30, *p*<.001, η_p_^2^ = .45). Specifically, the N2 peak was centrally distributed in children (Cz>FCz>Fz), but was frontally distributed in adults (Fz>FCz>Cz). The peak latency of the negativity observed in the Nogo – congruous difference waveform significantly decreased with age (*F*(1, 24) = 7.18, *p* = .013, η_p_^2^ = .23). Additionally, peak amplitude also decreased with age, although this effect was marginally significant (*F*(1, 24) = 3.93, *p* = .059, η_p_^2^ = .14). As no significant N2 effect was observed for the incongruous – congruous difference ERP in the group of children, no analyses were conducted.

### Source Localization Results

**Figure 4:**
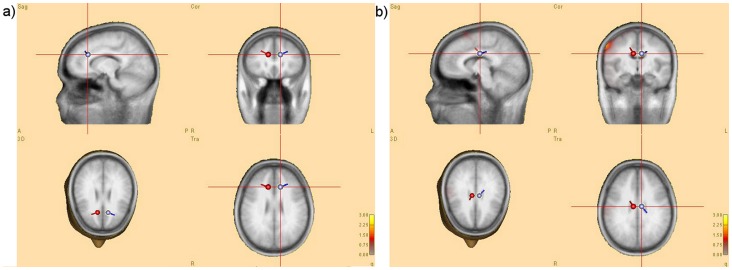
Source localisation analyses for (a) Nogo – congruous and (b) incongruous – congruous N2 effects in the adult group.

Source localization analyses were conducted on grand average ERP difference waveforms of each condition in the adult group (see Figure 4). In the Nogo condition, two symmetrical dipoles at Talairach coordinates (11.7, 27.1, 26.8) and (−11.7, 27.1, 26.8) accounted for 95.47% of the variance, mapping onto a more anterior region of the cingulate gyrus in each hemisphere [36], [37]. In the incongruous condition, two symmetrical dipoles at (8.1, −10.5, 28.8) and (−8.1, −10.5, 28.8) accounted for 95.17% of the variance in the ERP, mapping onto the cingulate gyrus in each hemisphere.

## Discussion

The results of this study showed that the N2 ERP changed in latency and topography between childhood and adulthood, and that the N2 effect was different following presentation of incongruous and Nogo stimuli in the two groups. The differences of amplitude, latency, and topography between conditions during development (as evidenced by the significant main effects and interactions of ANOVAs), as well as differences observed in the source localisation analyses conducted on the adult group, provide evidence of the separability of response inhibition and interference suppression [10], [13].

In the Nogo condition, the N2 effect was maximal at central scalp sites in children, but was maximal at frontal sites in adults. Additionally, source localisation found that the dipoles observed in adults are in frontal regions (see Figure 4). Previous research has found that neural activation associated with response inhibition becomes increasingly frontal with age through childhood development [11]. Frontal regions, including the anterior cingulate cortex, are commonly associated with behavioural performance on Go/Nogo tasks in adults [38], [39], and are one of the last regions of the brain to complete development [28], [40]. It appears that in the early stages of development of this region, children are more reliant upon more posterior regions of the brain in order to successfully inhibit responses [11], [41]. Additionally, a significant main effect of latency was observed. This may be explained by the large-scale myelination occurring throughout childhood and adolescence [30], [42], which is commonly thought to decrease ERP latency [43], [44]. A marginally significant decrease in amplitude was also observed between the two age groups, providing some support for previous research by Johnstone et al. [29], who found that N2 amplitude decreased with age, thought to be caused by fewer cognitive demands and increasingly efficient recruitment of relevant brain regions as individuals develop through childhood [31].

In the incongruous condition, there was no significant N2 effect in the group of children, whereas the effect was maximal at fronto-central sites in adults. Although an increase in the size of the effect from childhood to adulthood was hypothesised, it is somewhat surprising that no N2 effect at all was observed in children. It is possible that this lack of significant neural activation in children is caused by differences in the propagation of neural activation between childhood and adulthood. Previous neuroimaging research has reported that children display more diffuse activation of frontal regions, whereas the neural activation observed in adults is more focalised due to a gradual decrease in the number of synapses through childhood and adolescence, and an increase in the strength of connections between the remaining synapses during this time [45], [46]. Due to these weaker, more inefficient connections between synapses in children, it may be plausible that children ‘spread the load’ across a larger region of the brain, which results in less dense neural activation.

The results of this study could contribute to several avenues of future research, particularly in clinical settings. For example, examining the effects of traumatic brain injury (TBI) on response inhibition and interference suppression would provide further insight into the underlying neural generators of the two processes. Whilst some previous research [47], [48] has examined the effects of TBI on various cognitive tasks, no study has attempted to determine whether a differential deficit exists between these inhibitory subprocesses. Considering that previous research has highlighted clear differences in white matter integrity between TBI patients and control groups [48], it would be of particular interest to examine the latency of the N2 ERP, as an increased latency in TBI patients would provide a new perspective on the link between brain and behaviour in atypical groups.

Alternatively, examining differences between typically and atypically developing groups of children may be of benefit. Children born preterm, for instance, have been shown to be at increased risk of various cognitive deficits, including executive dysfunction [49], in addition to neurophysiological differences such as decreased brain volume [50], [51]. Research into differences between typically and atypically developing children can potentially provide further evidence of the separability of inhibitory subprocesses from a new perspective, strengthening theories of inhibition and its development [13].

In conclusion, the present study has added evidence from an electrophysiological perspective to the predominantly behavioural-based knowledge of the development of inhibitory processes [12], [13], [24]. Results from ERP analyses have reported topographical changes in both response inhibition and interference suppression, and latency and amplitude reductions in response inhibition. Additionally, source localisation analysis has provided evidence that the neural generators of response inhibition and interference suppression are distinct. Consistent with previous research, the current study suggests that the cingulate cortex is involved in, and highly important to, response inhibition and interference suppression respectively [52]–[56]. Furthermore, there are marked differences between age groups within each condition, providing neurophysiological evidence of different developmental trajectories of the two constructs. Theories of the development of inhibition and other higher-order cognitive functions (such as working memory) would greatly benefit from the integration of neuroscience with behavioural evidence.
